# A method for culturing Gram-negative skin microbiota

**DOI:** 10.1186/s12866-016-0684-9

**Published:** 2016-04-06

**Authors:** Ian A. Myles, Jensen D. Reckhow, Kelli W. Williams, Inka Sastalla, Karen M. Frank, Sandip K. Datta

**Affiliations:** Bacterial Pathogenesis Unit, Laboratory of Clinical Infectious Diseases, National Institute of Allergy and Infectious Diseases, National Institutes of Health, Bethesda, MD USA; Department of Laboratory Medicine, National Institutes of Health Clinical Center, Bethesda, MD USA

**Keywords:** Bacteriology, Skin, Microbiome, Culture techniques

## Abstract

**Background:**

Commensal Gram-negative (CGN) microbiota have been identified on human skin by DNA sequencing; however, methods to reliably culture viable Gram-negative skin organisms have not been previously described.

**Results:**

Through the use of selective antibiotics and minimal media we developed methods to culture CGN from skin swabs. We identified several previously uncharacterized CGN at the species level by optimizing growth conditions and limiting the inhibitory effects of nutrient shock, temperature, and bacterial competition, factors that may have previously limited CGN isolation from skin cultures.

**Conclusions:**

Our protocol will permit future functional studies on the influences of CGN on skin homeostasis and disease.

**Electronic supplementary material:**

The online version of this article (doi:10.1186/s12866-016-0684-9) contains supplementary material, which is available to authorized users.

## Background

A wealth of recent work has identified the microbiome as a major influence on human health and disease. Topographical surveys of the skin microbiota by 16S ribosomal RNA gene [1] and metagenomic shotgun [2] sequencing have highlighted the bacterial diversity found on the human body. These studies confirmed the previously appreciated prevalence of Gram-positive bacteria on the skin, including various *Staphylococcus* species and Actinobacteria such as *Propionibacterium* and *Corynebacterium*. Historically, low and inconsistent yields of Gram-negative bacteria from skin by culture led to the conclusion that Gram-negative species were absent or transient inhabitants of human skin [3, 4]. However, genomic approaches have identified Gram-negative bacteria as significant constituents of the skin biome, particularly at sites such as the antecubital fossa and volar forearm [1, 2]. Limitations in DNA analysis techniques have largely prevented species-level identification of these Gram-negative bacteria in the skin microbiome [1, 2, 5]. Here we describe novel methods to culture viable commensal Gram-negative (CGN) skin bacteria. This will allow species-level identification, whole genome sequencing, design of gene primers for enhanced molecular identification, and functional characterization of these bacteria and their role in skin homeostasis and disease.

## Methods

### Subject selection and sampling

Thirteen healthy adults, with no history of skin disease, were seen in our outpatient clinic. The participants were asked to refrain from bathing for the 24 h prior to their visits. For the nine participants who were amenable and able to return for repeat visits, isolation procedures were repeated to assess temporal consistency of culture findings. The antecubital fossa and volar forearm were selected as culture sites due to their propensity to contain CGN in published microbiome studies [1, 2] and their relevance as medically important sites for skin conditions such as atopic dermatitis.

### Gram-negative bacterial isolation

We first moistened two FloqSwabs (Copan, Brescia, Italy) in sterile phosphate buffered saline (PBS; Corning Cellgro, Corning, NY). Both swabs were simultaneously rubbed on the subject’s skin at the antecubital fossa and volar forearm vigorously for 15–30 s. One swab was placed into a 15 mL conical tube (Corning Life, Corning, NY) with 2 mL of sterile Hank’s balanced salt solution (HBSS; Sigma-Aldrich), vancomycin (300ug/mL), and amphotericin B (5ug/mL; Sigma-Aldrich, St. Louis, MO) to inhibit growth of Gram-positive bacteria and fungi. The remaining swab was placed into a 15 mL conical tube containing 2 mL of R2A broth (Teknova, Hollister, CA) with similar concentrations of vancomycin and amphotericin B. The tubes, with swabs left in place, were then incubated at 32 **°**C with constant shaking for 48–72 h under aerobic conditions before plating 100uL from each tube onto an R2A (Reasoner's 2A) agar plate (Remel, Lenexa, KS). R2A media is a relatively nutrient poor agar typically used for the isolation of slow-growing bacteria in potable water [6] (Additional file 1: Supplemental methods). Colonies were appreciable 48–72 h later. Individual colonies were then taken for species identification by mass spectrometry using matrix-assisted laser desorption/ionization-time of flight (MALDI-TOF) analysis. Bacterial protein extraction for MALDI-TOF MS using the BioTyper (v3.1, Bruker Daltonics Inc., Billerica, MA) was performed using previously described methods [7], instrument settings and calibration [8, 9]. BioTyper identification was supplemented by additional mass spectra profiles provided by several NIH developed databases [7, 10, 11]. Nine of the participants were swabbed at three different times, separated by at least 3 months over the course of a year and identical species were isolated on these sequential cultures (participants 1–2, 5–9, 12–13).

### Gram-positive bacterial isolation

Skin swabs obtained as described above were plated directly on blood agar or brain heart infusion agar (BHI) and incubated under aerobic conditions at 37 **°**C. *Staphylococcus aureus* was initially distinguished from other staphylococcal species by mannitol fermentation on mannitol salt agar. Speciation of suspected *S. aureus* isolates were confirmed by measuring coagulase activity (Fluka Chemicals, Switzerland).

### Consent

Written informed consent was obtained for all participants in this study. All participants were adults.

## Results and discussion

### Modification of media and temperature allows isolation of CGN from skin

**Fig. 1:**
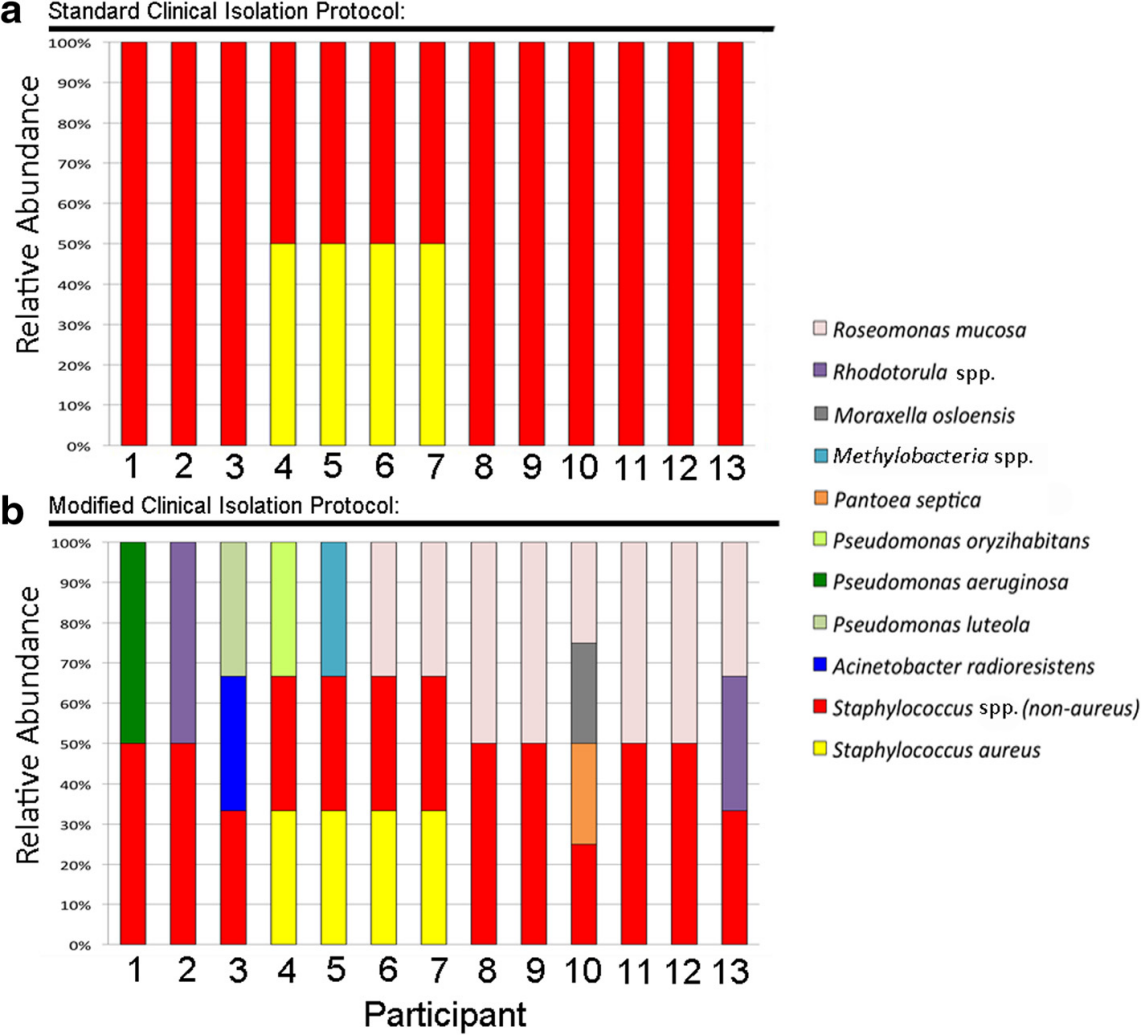
Culture protocol modifications allow isolation of Gram-negative skin microbiota. **a** Thirteen participants underwent standard clinical skin bacteria isolation using swabs plated onto blood, mannitol salt, BHI, chocolate, and MacConkey agar incubated at 37 **°**C. **b** The same participants underwent modified bacterial isolation as described. The relative abundance of cultured bacterial isolates from each participant is shown on the vertical axis. All bacteria were identified by MALDI-TOF mass spectrometry analysis, except *Staphylococcus* species were identified by characteristic growth on mannitol salt agar plates with positives confirmed by coagulase testing. For participants that were re-sampled, no discrepancies between initial and subsequent isolations were found

Standard culture techniques for Gram-negative bacteria from sources other than skin involve incubation at 37 **°**C using liquid media such as tryptic soy broth (TSB), or on solid media such as chocolate, blood, or MacConkey [12]. We attempted to culture skin bacteria from thirteen participants by plating forearm swabs onto 5 % sheep blood, mannitol salt, BHI, chocolate, and MacConkey agar incubated at 37 **°**C. Our use of these techniques readily isolated staphylococcal species from multiple healthy volunteers but failed to culture any Gram-negative isolates, even with the use of a Gram-negative selective agar such as MacConkey (Fig. 1a).

**Fig. 2:**
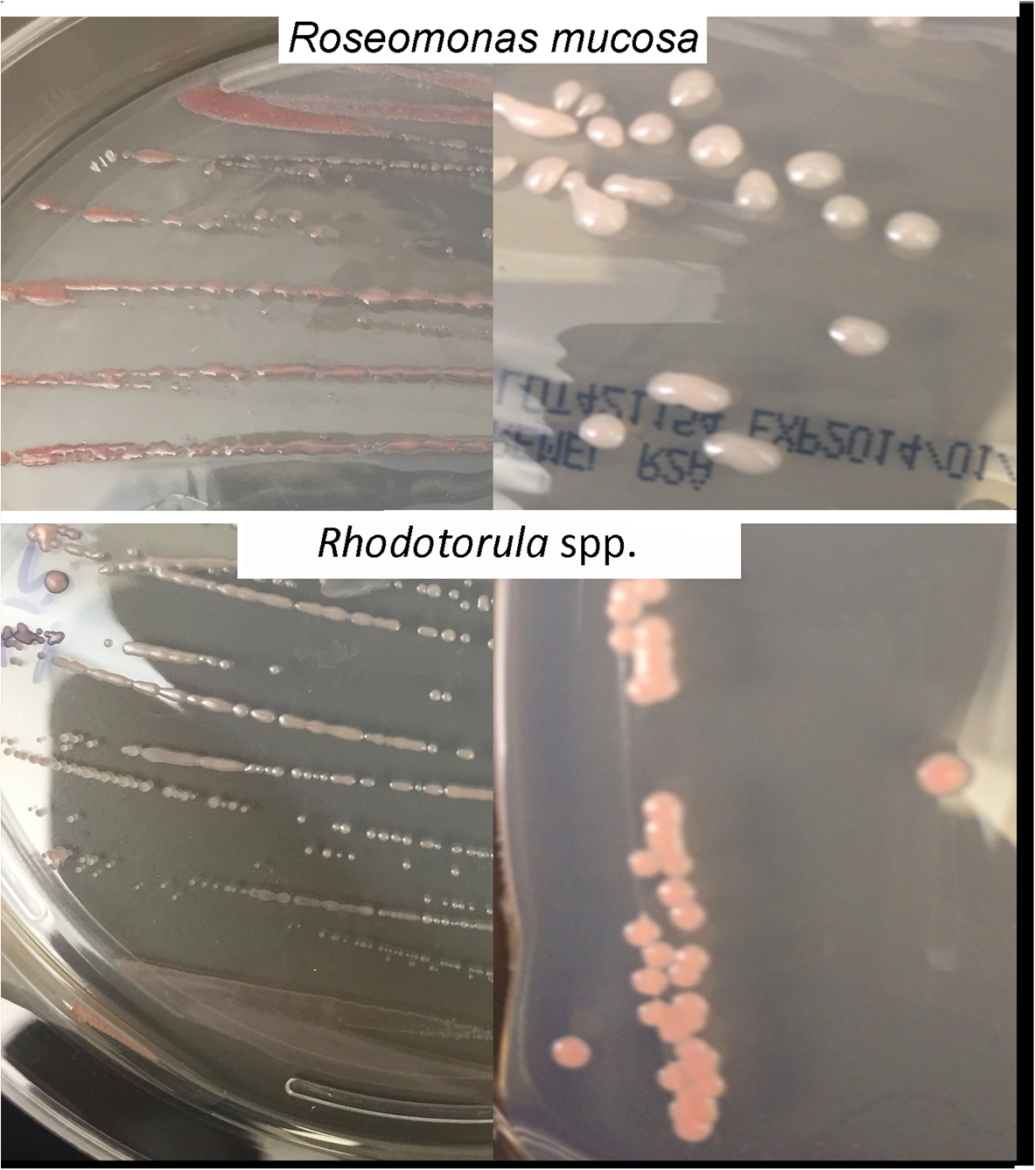
Colony morphology for *Roseomonas mucosa* and *Rhodotorula* spp. Colony morphology for two different strains of *Roseomonas mucosa* (*top*) and *Rhodotorula* spp. *(bottom; R. mucilaginosa, right*; *R. minuta/slooffiae*, *left*) streaked linearly (*left*) or with four-quadrant technique (*right*) on R2A agar

The use of our modified Gram-negative bacteria isolation protocol (see Methods) yielded several Gram-negative species from the forearm skin of healthy volunteers (Fig. 1b). The predominant Gram-negative bacterium isolated by our methods was *Roseomonas mucosa,* a member of the alphaproteobacteria class. For two volunteers (2 and 3), no growth was seen from R2A broth, but indicated species were isolated from the HBSS tube. Other species isolated included the gammaproteobacteria *Pseudomonas aeruginosa, Pseudomonas luteola, Pseudomonas oryzihabitans, Acinetobacter radioresistens, Pantoea septica*, and *Moraxella osloensis*. Swabs from one individual grew *Methylobacterium* species (alphaproteobacteria). These results are consistent with prior reports using phylogenetic and metagenomic sequence analysis [1, 2]. Two subjects grew yeast, *Rhodotorula* spp. (*R. mucilaginosa* and *R. minuta/slooffiae*), despite the presence of amphotericin B during culture, including one healthy subject from whom no Gram-negatives were cultured (identified by MALDI-TOF followed by sequencing of ITS region as described previously [8]). Colonies of *Roseomonas mucosa* and both *Rhodotorula* spp. had some initial morphological similarities that may make discernment difficult if each species is viewed in isolation (Fig. 2), but they were readily distinguished by Gram-staining.

### CGN from skin have more protracted growth curves than Gram-positive isolates

**Fig. 3:**
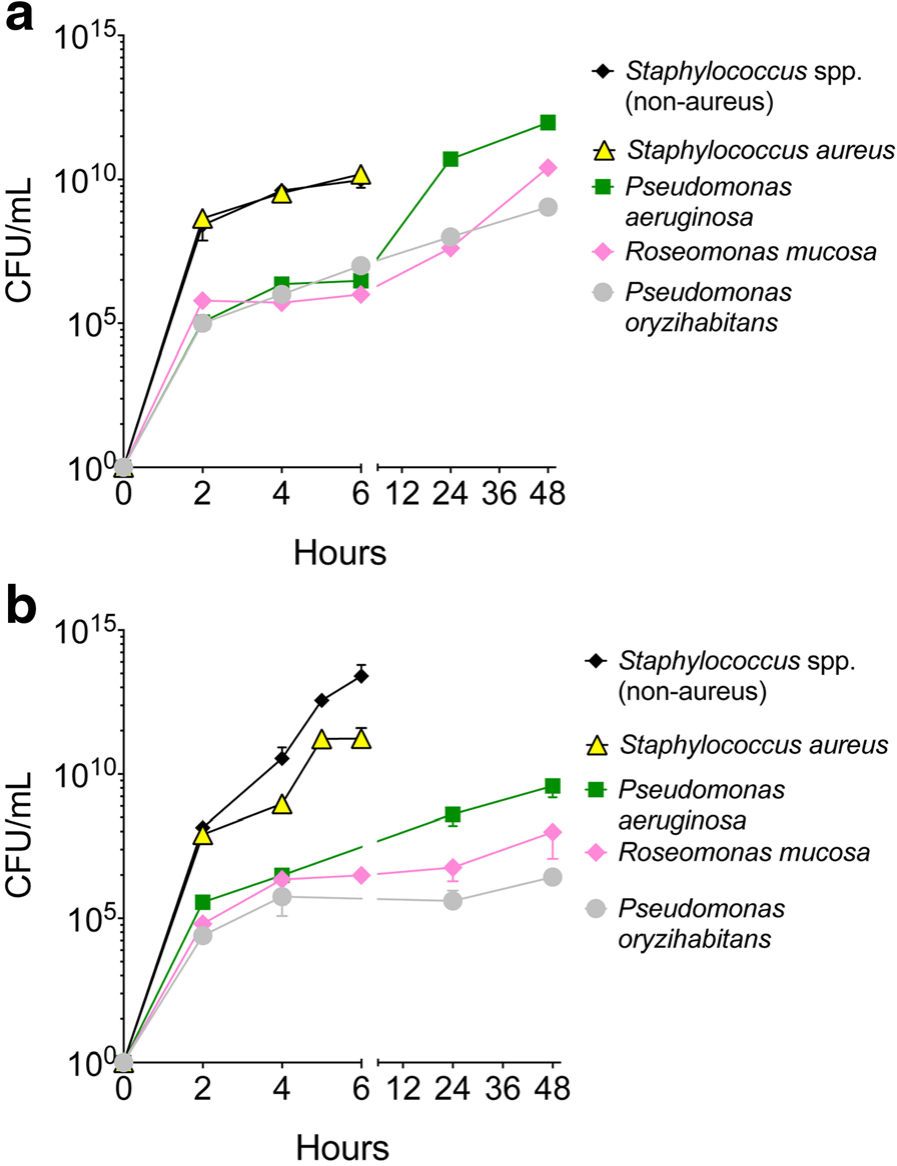
Growth curves for select commensal Gram-negative and *Staphylococcus* species. A single colony of bacteria was added to liquid media at time zero for all indicated isolates. Colony forming units (CFU) assessed by serial dilutions at indicated time points. **a** Growth performed at 32 **°**C, in R2A broth for both CGN and staphylococcal species for direct comparison of growth kinetics. **b** Comparison of optimized growth kinetics; CGN cultured at 32 **°**C in R2A broth, *Staphylococcus* species cultured at 37 **°**C in TSB. Data are representative of two or more independent experiments and depicted as mean + SD. Each species represented by 1–3 clinical isolates

The growth kinetics of the CGN isolates were variable, but most showed completion of exponential growth by 4-6 h (Fig. 3a–b). Staphylococcal strains reached a nearly five-log higher CFU total by six hours in either nutrient-poor or nutrient-rich broth (Fig. 3a–b). Thus, one explanation for prior failures to culture CGN could involve the ability of staphylococci and other robustly growing organisms to outpace the growth of CGN, making inclusion of antimicrobials, such as vancomycin and amphotericin B, critical for the isolation of CGN flora.

### CGN from skin are slower growing than Gram-positive isolates

After incubation of skin swabs in HBSS or R2A, the initial isolation of *R. mucosa*, *P. septica*, and *M. osloensis* was only successful when using R2A plates (Table 1). Maximal yield was obtained when these isolates were incubated at 32 **°**C, a temperature more reflective of the skin surface than 37 **°**C [13]. Initial isolation of the *Pseudomonas* species were additionally successful on blood agar if incubated at 32 **°**C and on chocolate agar at both 32 **°**C and 37 **°**C (Table 1). Once our initial swabs yielded viable bacterial colonies, these could then be subsequently grown on many nutrient rich agars (Table 1). However, our *R. mucosa* strains failed to grow on chocolate or MacConkey agar, even when plating at a 1e8 or greater inoculum. Taken together, these findings suggest that previous failures to consistently isolate CGN bacteria may have been due to staphylococcal competition, the use of nutrient rich media, and choice of 37 **°**C incubation. However, once a high enough pure inoculum has been collected, limitations in media and temperature can be overcome on select media.

**Table 1:** Solid media type influences isolation of Gram-negative skin microbiota. (Upper) Growth results from initial swab collected from participant on various plates and at either 32 or 37 **°**C using 4-quadrant streaking system. (Lower) Growth yields after inoculum isolated on R2A was re-plated under indicated conditions. Increasing amounts of bacterial growth are semi-quantitatively indicated as none (--), or as apparent only in the 1^st^ quadrant (+), or into the 2^nd^ (++) or 3^rd^ (+++) streaked quadrant at 72 h. Data are representative of three independent experiments for each species. Each species represented by 1–3 clinical isolates

	From Initial Swab									
	Blood Agar		BHI		Chocolate		MacConkey		R2A	
Bacteria	32	37	32	37	32	37	32	37	32	37
*R. mucosa*	--	--	--	--	--	--	--	--	+++	+
*P. septica*	--	--	--	--	--	--	--	--	+++	+
*M. osloensis*	--	--	--	--	--	--	--	--	+++	+
*P. aeruginosa*	++	--	--	--	++	+	++	+	+++	+
*P. oryhabiztans*	+	--	--	--	++	+	--	--	+++	+
*P. luteola*	+	--	--	--	++	+	--	--	+++	+
	After Isolation									
	Blood Agar		BHI		Chocolate		MacConkey		R2A	
Bacteria	32	37	32	37	32	37	32	37	32	37
*R. mucosa*	++	++	+	+	--	--	--	--	+++	++
*P. septica*	++	++	++	++	++	++	+	+	+++	++
*M. osloensis*	++	++	++	++	+	+	--	--	+++	++
*P. aeruginosa*	++	--	++	++	++	+	+	++	+++	++
*P. oryhabiztans*	++	--	++	++	++	+	++	+	+++	++
*P. luteola*	++	++	++	++	++	+	++	+	+++	++

### CGN from the skin showed evidence of nutrient shock

Failure of bacteria to grow in nutrient rich environments has been termed ‘nutrient shock’. This has been proposed as a mechanism to explain the presence of ‘viable but nonculturable bacteria’ seen by staining techniques, and the detection of DNA signatures for bacteria that are nonculturable. Recent work has quantified evidence of nutrient shock [14]. We performed similar analysis by first growing *R. mucosa*, *P. septica*, and *M. osloensis* in R2A broth for 48 h. Then each isolate was diluted 1:50 into either TSB (nutrient rich) or R2A (nutrient poor) and plated on R2A agar immediately and again after 48 h of incubation at 32 **°**C. Dilution into TSB led to an 80 % reduction in CFU after 48 h for *R. mucosa* and *M. osloensis* compared to dilution into R2A (Additional file 1: Figure S1). In contrast, *P. septica* was only affected at the earlier time point. While the mechanisms contributing to nutrient shock are unclear, our data support the claim that certain Gram-negative bacteria may not be easily recovered on select nutrient rich agar.

## Conclusions

To our knowledge, our modified culture methods are the first to consistently isolate Gram-negative bacterial commensals from human skin. Our culture methods yielded up to three species of CGN from a single individual and many individuals yielded just one species. It is likely that additional species not amenable to our culture methods exist and remain to be identified. Indeed, the microbiome literature on 16S ribosomal DNA signatures suggests 2-5 classes of Gram-negative bacteria are present on the skin of a given individual [1, 15], although the number of different species represented within these classes is unknown. Consistent with our data, sophisticated analyses of metagenomic shotgun sequencing of the skin microbiome has identified *Pseudomonas* and *Roseomonas* species as well as other Gram-negative genera, although limitations in reference genome databases and pooled analysis across individuals still prevented more exact determination of bacterial burden at the species level in a given individual [2]. Our data are also limited by the focus on forearm skin and exclusion of anaerobic culture conditions. For example, in a subset of patients with acne, 16S ribosomal DNA data from facial swabs detected *Escherichia coli* [16], a bacterium we did not culture with our techniques. However, successful culture of even select Gram-negative microbiota using our new methodologies will allow whole genome sequencing and characterization of these specific colonizing strains at the biochemical, metabolic, and functional level that may lend insight into the interactions of these bacteria with their host.

### Ethics approval and consent to participate

All clinical isolates were processed in a de-identified fashion under the National Institute of Allergy and Infectious Diseases Internal Review Board (IRB)-approved trial NCT02262819. All participants signed informed consent prior to isolate collection.

### Consent for publication

Not applicable.

### Availability of data and material

Supplemental information is available online. See Additional file 1 section below for details.

## Additional file

Additional file 1:
**Figure S1.** Growth of select commensal Gram-negative species after acute nutrient change. Data depicts the impact on growth for transferring cultures of *R. mucosa*, *P. septica*, *M. osloensis* from low nutrient broth to high nutrient broth. **Supplemental Detailed Methods.** Detailed protocol of Commensal Gram Negative Isolation. Step by step protocol used in CGN isolation from skin. (DOCX 707 kb)

## Abbreviations

BHI: blood heart infusion
CGN: Commensal Gram-negative
MALDI-TOF: matrix-assisted laser desorption/ionization-time of flight
R2A: Reasoner's 2A
TSB: tryptic soy broth
