# Supplementary material for: A method for culturing Gram-negative skin microbiota
Source: BMC Microbiol. 2016 Apr 6;16:60. doi: 10.1186/s12866-016-0684-9 (PMC4823881; doi:10.1186/s12866-016-0684-9)
Supplement: Additional file 1: — Figure S1. Growth of select commensal Gram-negative species after acute nutrient change. Data depicts the impact on growth for transferring cultures of R. mucosa, P. septica, M. osloensis from low nutrient broth to high nutrient broth. Supplemental Detailed Methods. Detailed protocol of Commensal Gram Negative Isolation. Step by step protocol used in CGN isolation from skin. (DOCX 707 kb) [file 12866_2016_684_MOESM1_ESM.docx]

Additional file 1


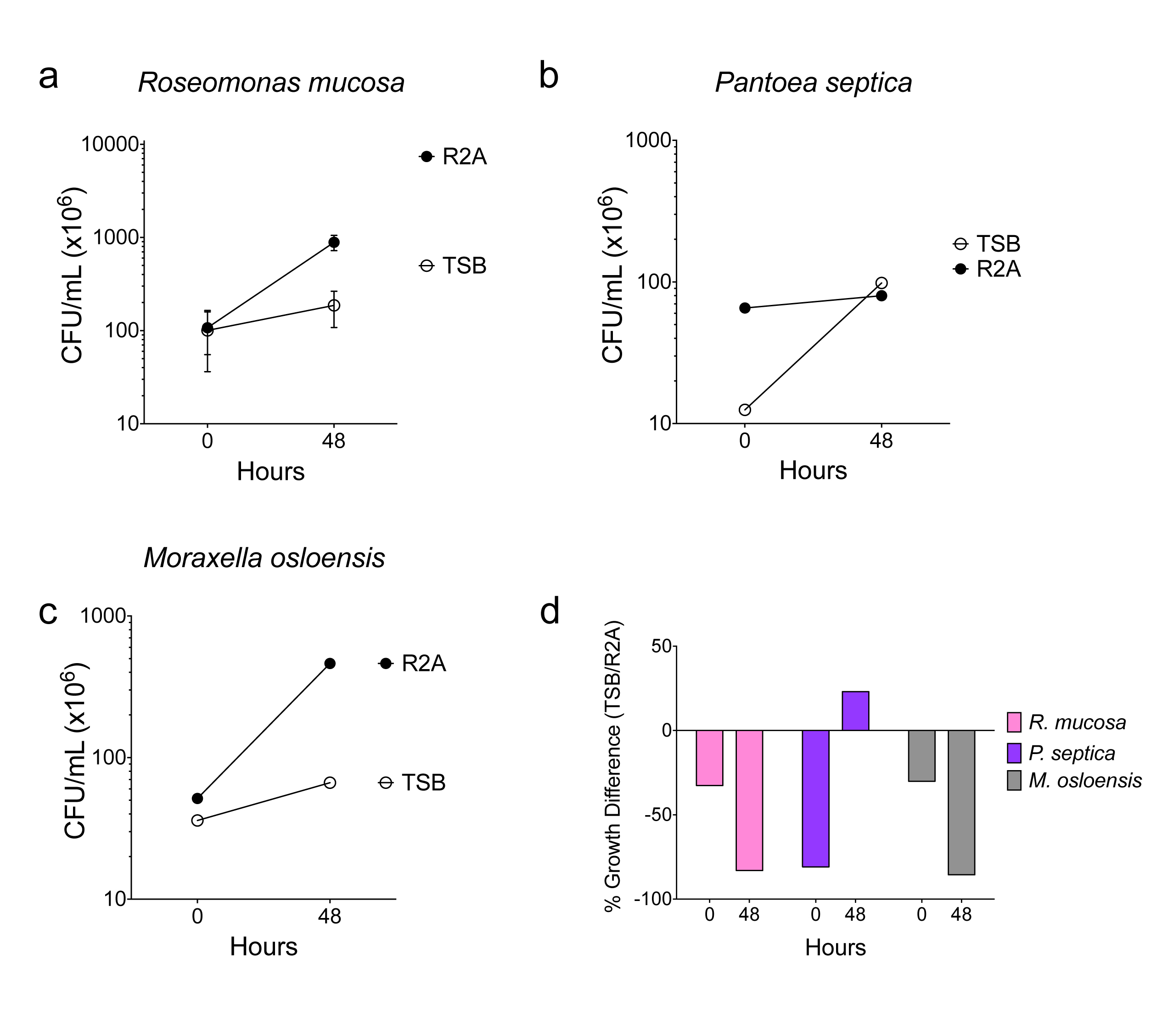


**Figure S1. Growth of select commensal Gram-negative species after acute nutrient change.** Liquid cultures of *R. mucosa*, *P. septica*, and *M. osloensis* were incubated for 48 hours in R2A broth at 32**°**C. 100mcL of this culture was diluted into 5mL of R2A or TSB. A sample taken immediately (0 hours) or after 48 hours of culture at 32**°**C was serial diluted onto R2A plates to determine colony forming units (CFU). CFU per mL shown for *R. mucosa* (a), *P. septica* (b), and *M. osloensis* (c). (d) Percent of CFU yield from TSB culture as compared to yield from R2A culture. Data are either combined from two experiments using two isolates per experiment (a) or are representative of two independent experiments with one clinical isolate each (c-d). Data depicted as mean + SD.

**Supplemental Detailed Methods:**

**Needed at time of collection:**

1. 6 swabs (FLOQSwabs 220250).
2. 15mL conical with ~2mL of PBS.
3. 2x 15mL conical tubes with 2mL of TSB each.
4. 2x 15mL conical tubes with 5mL HBSS+antibiotic broth each.
5. 2x 15mL conical tubes with 5mL R2A+ antibiotic broth each.

For antimicrobials:

Vancomycin: final concentration 300ug/5mL

Amphotericin B: final concentration 25ug/5mL

**Collection (day 1):**

1. Dip 3 swabs in PBS.
2. Swab each forearm with 3 swabs at a time, vigorously rub on skin.
3. Put one swab into R2A broth, one into HBSS, final into TSB tubes.
   1. R2A media typical contents: 0.5g/L of proteose peptone, casamino acids, yeast extract, dextrose, soluble starch; 0.3g/L dipotassium phosphate, sodium pyruvate; 0.05g/L magnesium sulfate; +/- 15g/L agar for solid media.
4. Repeat for opposing arm.

**Processing (day 1):**

1. Vortex each on medium speed for 30 seconds.
2. Remove swabs from TSB tubes (Leave swab in R2A and HBSS).
3. Put R2A broth and HBSS in the 32 **°**C incubator on constant stir.
4. Plate 100uL of each TSB stock collection on a mannitol salt agar (MSA) plate, place plate in 37**°**C.
5. Place remaining TSB in 37**°**C incubator overnight. If there is no growth on the MSA plates, dilute overnight growth (10e-3 to 10e-10) and re-plate the next day onto MSA.

**Collect *Staphylococcus* species (day 2):**

1. Collect yellow colonies from MSA plate. These are presumed *Staphylococcus aureus* isolates; speciation can be further verified by standard techniques such as coagulase testing.
2. Collect white or non-yellow colonies from MSA plate. These are presumed *Staphylococcus* spp (non-aureus) isolates; speciation can be further verified by standard techniques.

**Plate Gram-negative species (day 3):**

1. Take 100uL of the R2A and HBSS culture and plate on R2A plate after serial dilution (from 10e-3 to 10e-10).

**Collect Gram-negative species (day 4-6):**

Collect individual colonies and sub-culture or type as needed
